# Chmp 1A is a mediator of the anti-proliferative effects of All-trans Retinoic Acid in human pancreatic cancer cells

**DOI:** 10.1186/1476-4598-8-7

**Published:** 2009-02-12

**Authors:** Jing Li, Brandon Orr, Kayla White, Natalia Belogortseva, Richard Niles, Goran Boskovic, Hanh Nguyen, Ava Dykes, Maiyon Park

**Affiliations:** 1 Department of Biochemistry and Microbiology, Joan C. Edwards School of Medicine, Marshall University, Huntington WV 25755, USA; 2 Genomic Core, Joan C. Edwards School of Medicine, Marshall University, Huntington WV, USA; 3 Imaging Core, Joan C. Edwards School of Medicine, Marshall University, Huntington WV, USA

## Abstract

**Background:**

We recently have shown that Charged multivesicular protein/Chromatin modifying protein1A (Chmp1A) functions as a tumor suppressor in human pancreatic tumor cells. Pancreatic cancer has the worst prognosis of all cancers with a dismal 5-year survival rate. Preclinical studies using ATRA for treating human pancreatic cancer suggest this compound might be useful for treatment of pancreatic cancer patients. However, the molecular mechanism by which ATRA inhibits growth of pancreatic cancer cells is not clear. The objective of our study was to investigate whether Chmp1A is involved in ATRA-mediated growth inhibition of human pancreatic tumor cells.

**Results:**

We performed microarray studies using HEK 293T cells and discovered that Chmp1A positively regulated Cellular retinol-binding protein 1 (CRBP-1). CRBP-1 is a key regulator of All-trans retinoic acid (ATRA) through ATRA metabolism and nuclear localization. Since our microarray data indicates a potential involvement of Chmp1A in ATRA signaling, we tested this hypothesis by treating pancreatic tumor cells with ATRA *in vitro*. In the ATRA-responsive cell lines, ATRA significantly increased the protein expression of Chmp1A, CRBP-1, P53 and phospho-P53 at serine 15 and 37 position. We found that knockdown of Chmp1A via shRNA abolished the ATRA-mediated growth inhibition of PanC-1 cells. Also, Chmp1A silencing diminished the increase of Chmp1A, P53 and phospho-P53 protein expression induced by ATRA. In the ATRA non-responsive cells, ATRA did not have any effect on the protein level of Chmp1A and P53. Chmp1A over-expression, however, induced growth inhibition of ATRA non-responsive cells, which was accompanied by an increase of Chmp1A, P53 and phospho-P53. Interestingly, in ATRA responsive cells Chmp1A is localized to the nucleus, which became robust upon ATRA treatment. In the ATRA-non-responsive cells, Chmp1A was mainly translocated to the plasma membrane upon ATRA treatment.

**Conclusion:**

Collectively our data provides evidence that Chmp1A mediates the growth inhibitory activity of ATRA in human pancreatic cancer cells via regulation of CRBP-1. Our results also suggest that nuclear localization of Chmp1A is important in mediating ATRA signaling.

## Background

Retinoids are natural or synthetic derivatives of vitamin A. Nuclear receptors and cellular binding proteins are involved in mediating the biological effects of retinoids [1-4]. The active form of retinoids (ATRA or 9-*cis*-RA) interacts with their nuclear receptors, retinoic acid receptors (RAR α, β, and γ), and retinoic × receptors (RXR α, β, and γ). All-trans retinoic acid (ATRA) is one of the most physiologically active members of the retinoid family. By binding to its receptor RAR, ATRA exercises a broad spectrum of biological effects such as proliferation and differentiation [5]. Because of anti-proliferative effects of ATRA, it has been used as a therapeutic and/or preventive agent in certain cancers such as promyelocytic leukemia [6].

There are two cellular retinol-binding proteins; CRBP-I and II [7]. CRBP-1 is required for biosynthesis and metabolism of retinoic acid. Cellular retinol-binding protein I (CRBP-I) controls ATRA activity by presenting the retinol to various enzymes for retinoic acid synthesis [7-9]. CRBP-I protein expression was previously shown to correlate with tumor growth [8,10]. P53, a known tumor suppressor, is mutated in most tumors including pancreatic cancer. The mutation of P53 causes further mutation of the P53 gene itself, an increase in an ubiquitin-dependant degradation of P53 mediated by MDM2 [11], and an inactivation of P53 [12]. P53, located at chromosome 17, has a DNA binding domain and acts as a "genome gatekeeper" [13]. The inherited loss of one copy of an allele of P53 [14-16] usually results in several independent tumors in early adulthood (Li-Faumeni syndrome) [17].

Chmp1A (Charged multivesicular protein 1A/Chromatin modifying protein 1A) belongs to the class E family of Vps and is also called Vps 46p [18,19]. Chmp1A was shown to physically associate with the multivesicular sorting protein, SKD1/VPS4 (Vacuolar protein-sorting 4), with AMSH, an endosome-associated ubiquitin isopeptidase, and with VPS4 ATPases [18]. Chmp1A localizes at the endosomes, where it functions in the formation and sorting of multivesicular bodies (MVBs) [18]. Chmp1A was also reported to silence gene activation by interacting with a transcriptional repressor Polycomb-group (PcG) protein, BMI1 [19-21]. We have recently shown that Chmp1A is a novel tumor suppressor, especially in the pancreas. Chmp1A mRNA and protein was reduced and/or altered (protein) in various human pancreatic tumors. Stable over-expression of Chmp1A in PanC-1 cells resulted in cell growth inhibition and tumor xenograft inhibition, respectively. In contrast, silencing of Chmp1A in PanC-1 cells resulted in the elevation of cell and xenograft tumor growth. Over-expression of Chmp1A strongly increased the protein level of P53 and phospho-P53 indicating that Chmp1A regulates tumor growth potentially through a P53 signaling pathway [22].

Pancreatic cancer has the worst prognosis of all cancers with a dismal 5-year survival rate [23,24]. ATRA alone or in combination with other chemotherapeutic reagents has been successful in treating tumors [25-27]. Preclinical studies using ATRA for treating human pancreatic cancer suggest this compound might be useful for treatment of patients in pancreatic cancer. However, the molecular mechanism by which ATRA inhibits growth of pancreatic cancer cells is not clear. This paper focuses on the role of Chmp1A in ATRA mediated growth inhibition. The objective of our study was to investigate whether Chmp1A expression and/or localization is essential for ATRA mediated growth inhibition of human pancreatic tumor cells.

## Materials and methods

### Antibodies and chemicals

Rabbit polyclonal antibody against Chmp1A was generated in our laboratory by using recombinant Chmp1A protein (Belogortseva and Park, unpublished) and successfully used for the previous studies [22]. Other antibodies were purchased from commercial sources: rabbit polyclonal antibodies against P53 (Cell Signaling) and Phospho-P53 (Cell Signaling); rabbit polyclonal antibodies against phospho-specific P53 at Ser37 (Cell Signaling), and mouse monoclonal antibody phospho-specific P53 at Ser15 (Cell Signaling); mouse monoclonal antibodies against Gapdh (Cell Signaling), and rabbit polyclonal antibody against CRBP-1 (Abcam). Goat anti-rabbit/mouse HRP conjugated secondary antibody was purchased from Chemicon. All Trans Retinoic Acid was purchased from Fluka. Puromycin was purchased from Invitrogen. All other chemical reagents were purchased from Sigma, unless otherwise described.

### Cell Culture

All cell lines were obtained from American Type Culture Collection (Manassas, VA). PanC-1 (human pancreatic ductal tumor cells, poorly differentiated), CRL-2151 (mouse acinar tumor cells), and HEK 293T (human embryonic kidney, CRL-11268) cells were cultured in Dulbecco's modified Eagle's medium containing 10% fetal bovine serum (FBS, Gibco). Capan-2 (human pancreatic ductal tumor, well differentiated) cells were cultured in McCoy with 10% fetal bovine serum. All cell culture assays were performed at 37°C under 5% CO_2_.

### RT-PCR (reverse transcriptase-PCR)

HEK 293Tcells (CRL-11268) were transiently transfected with empty CS2+ (control) or Chimp1A-CS2+ plasmid using Plus and Lipofectamine reagent (Invitrogen). Total RNA was isolated 18 hours post-transfection using Trizol reagent (Invitrogen). Quality and quantity of isolated RNA was analyzed using Bioanalyzer (Agilent). RT-PCR (reverse-transcriptase-PCR) was performed using Titan one tube RT-PCR system that was purchased from Roche. For each reaction, 500 ng of total RNA was reverse transcribed and used to amplify Chmp1A and CRBP-1. PCR products were separated on 1.5% agarose gel containing Ethidium bromide. The forward and backward primers used to detect Chmp1A was: 5'-GAGACAGCGGGTCCGTAAC-3', and 5'-ACCTGGGCCATATTCTTGGT-3'. CRBP-1 primer used in this experiment was described in Arapshian, A., et al., [28]. The cycling parameter for amplifying PCR products was: 94° for 2 min, 10 cycles of (94° for 30 second, 48° for 30 second, 68° for 1 min), followed by 15, or 18 additional cycles of (94° for 30 second, 48° for 30 second, 68° for 1 min (+5 second per cycle), and last step is 68° for 7 min.

### ATRA treatment

PanC-1 and Capan-2 cells were seeded at 350,000 and 300,000 cells per 10 cm plate respectively. CRL-2151 cells were seeded at higher density since these cells are much smaller than Capan-2 or PanC-1 cells. The next day, cells were replaced with fresh media containing either vehicle (DMSO) or ATRA (20 μM final concentration). ATRA treated cells were kept in the dark since ATRA is light sensitive. Every other day, cells were replaced with fresh media containing either DMSO or ATRA. The cells were cultured for up to 6 days for the following experiments. For growth assays we maintained and treated cells in either regular or charcoal stripped FBS supplemented media. Charcoal stripped FBS was purchased from Invitrogen. We have received similar results with either regular or charcoal stripped serum. The data presented here was from the results we obtained with media supplemented with charcoal stripped serum.

### Generation of stable Chmp1A knockdown clones of PanC-1 cells

PanC-1 cells were cultured in DMEM media supplied with 10% FBS. RNAintro™ pSM2 retroviral vector (Open Biosystems) was used to sub-clone non-silencing or Chmp1A shRNAs. The Chmp1A shRNA sequence was designed using online software from Open Biosystems. This vector contains a puromycin-resistant marker site for positive colony selection. The specificity of Chmp1A shRNA was verified by transient transfection using Arrest-In transfect reagent (Open Biosystem) followed by Western blotting. To generate stable cell lines, shRNAs targeted to Chmp1A or non-silencing control were transfected into PanC-1 cells. Stable transfectants were selected in the presence of 2 ug/ml puromycin (Invitrogen), whose working concentration was determined by kill curve. Cells derived from these transfectants were used for Western blotting to confirm the decrease of Chmp1A protein expression. Chmp1A knockdown stable Panc-1 cells were maintained in DMEM media supplied with 10% FBS containing 1ug/ml puromycin.

### Stable over-expression of Chmp1A in PanC-1 cells

Tet-On advanced inducible gene expression system was used to generate conditional stable clones of PanC-1 cells (Clontech). Transfection was performed with CalPhos™ Mammalian Transfection Kit (Clontech) according to the manufacturer's instructions. Detailed protocol was described in Li et al., [22].

### Transient over-expression of Chmp1A in CRL-2151 cells

Equal numbers of CRL-2151 cells were seeded in 10 cm dishes one day before transfection. Next day, 5 ug of control CS2+ or Chmp1A-CS2+ plasmid was transiently transfected using Plus and Lipofectamine reagent following the company's instruction (Invitrogen). Cell numbers were counted the following three days, three dishes per each day for control or Chmp1A over-expression, respectively. After cell counting, cells were lysed for protein isolation. Protein concentration was determined using BCA reagent (Pierce) for Western blot analysis.

### Western blot analysis

**Figure 1:**
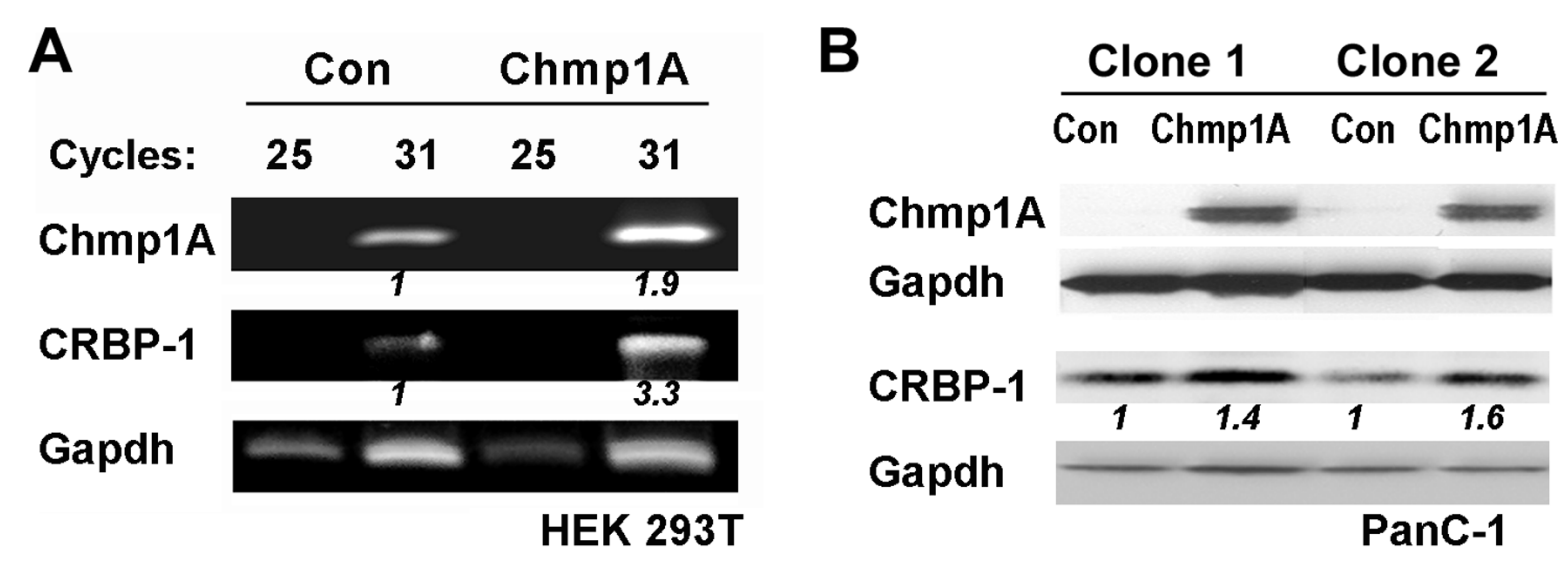
**Chmp1A over-expression positively regulates CRBP-1**. (A) HEK 293T cells were transfected with either empty CS2+ or Chmp1A-CS2+ plasmid. 18 hours after transfection cells were processed for total RNA isolation. Reverse transcriptase PCR (RT-PCR) indicates that Chmp1A and CRBP-1 transcripts were increased 1.5 and 3.3 folds respectively at 31 cycles compared to control. Gapdh was used as RT-PCR control. (B) Chmp1A expression was induced by the addition of doxycycline to the media in two independent stable clones of PanC-1 cells. Cells were lysed two days after the doxycycline supplement and processed for Western blot analysis. Chmp1A and CRBP-1 protein was increased upon Chmp1A over-expression. Con: Control

**Figure 2:**
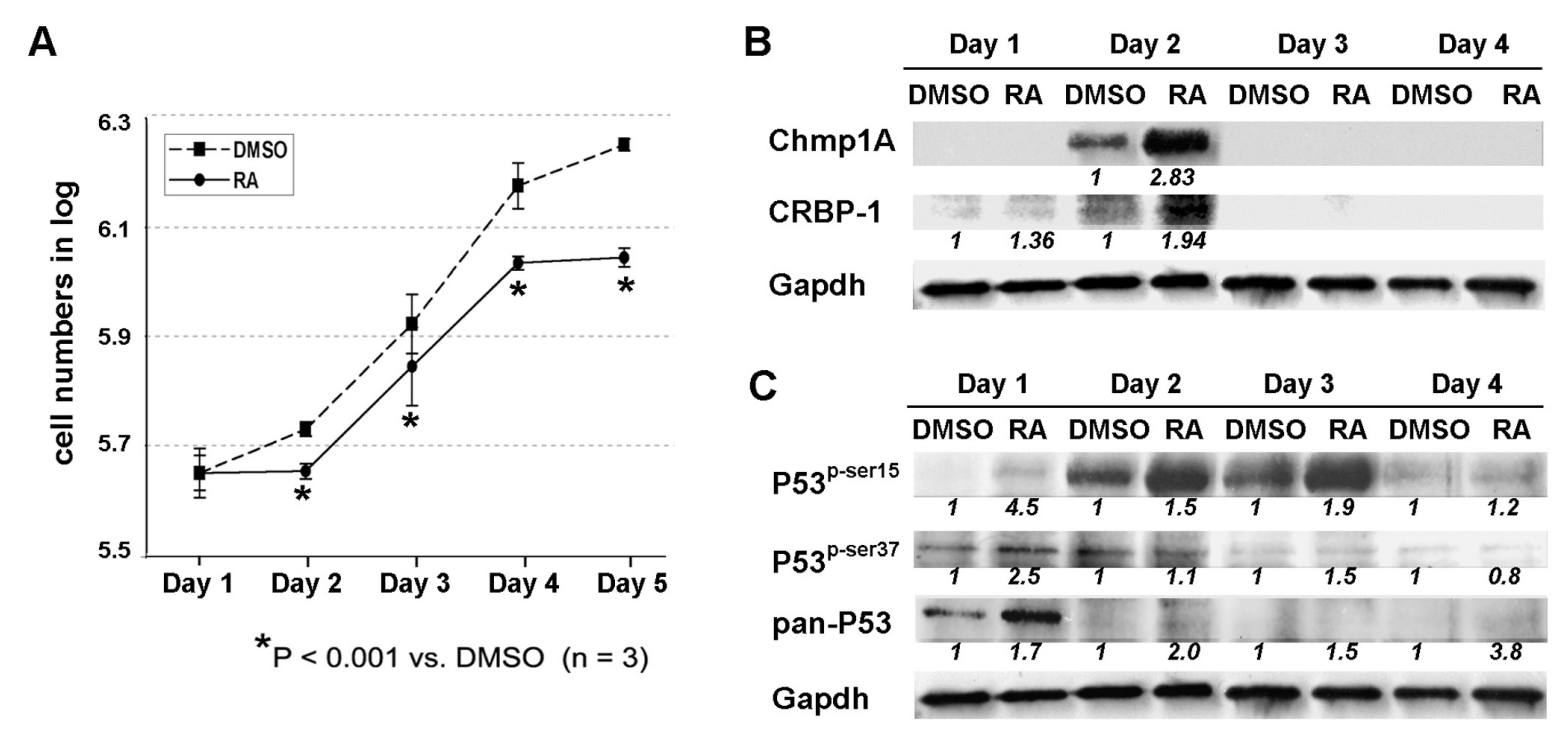
**The growth inhibition of Capan-2 cells by ATRA was accompanied by an increase in the protein level of Chmp1A, CRBP-1, P53, and phospho-P53**. (A) Capan-2 cells were counted and equal numbers of cells were seeded in 10 cm tissue culture dishes. Next day, cells were treated with control DMSO or ATRA. The following 5 Days, cells were counted and plotted for the graph. Dashed line with square represents DMSO treated and straight line with circle represents ATRA treated. On Day 1 the cells did not show any difference in growth. However, the growth was inhibited significantly (based on P-value) by ATRA treatment from Day 2 on. (B, C) The cells were processed for Western blot analysis following lysis. Notice the increase of protein level of Chmp1A, CRBP-1, P53 and phospho-P53 at serine 15 and 37 upon ATRA treatment.

**Figure 3:**
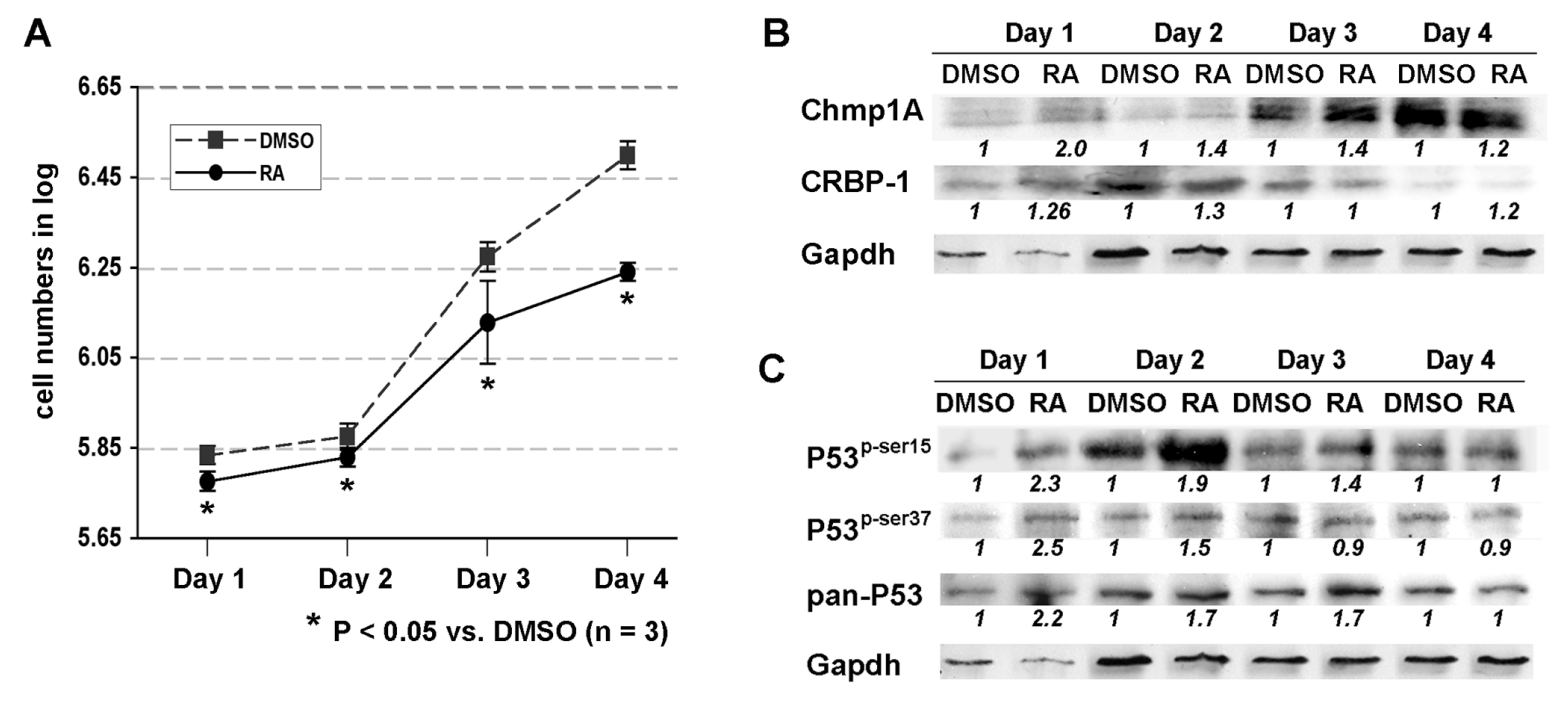
**The growth inhibition of PanC-1 cells by ATRA was accompanied by an increase of Chmp1A, CRBP-1, P53 and phospho-P53 protein expression**. (A) PanC-1 cells were counted and equal numbers of cells were seeded in 10 cm tissue culture dishes. Next day, cells were treated with DMSO or ATRA. The following 4 days, cells were counted and plotted for the graph. Dashed line with square represents DMSO treated and straight line with circle represents ATRA treated. P value indicates a significant growth inhibition by ATRA treatment. (B, C) The cells were processed for Western blot analysis after lysis. Notice the increase of Chmp1A and CRBP-1, P53 and phospho-P53 upon ATRA treatment.

**Figure 4:**
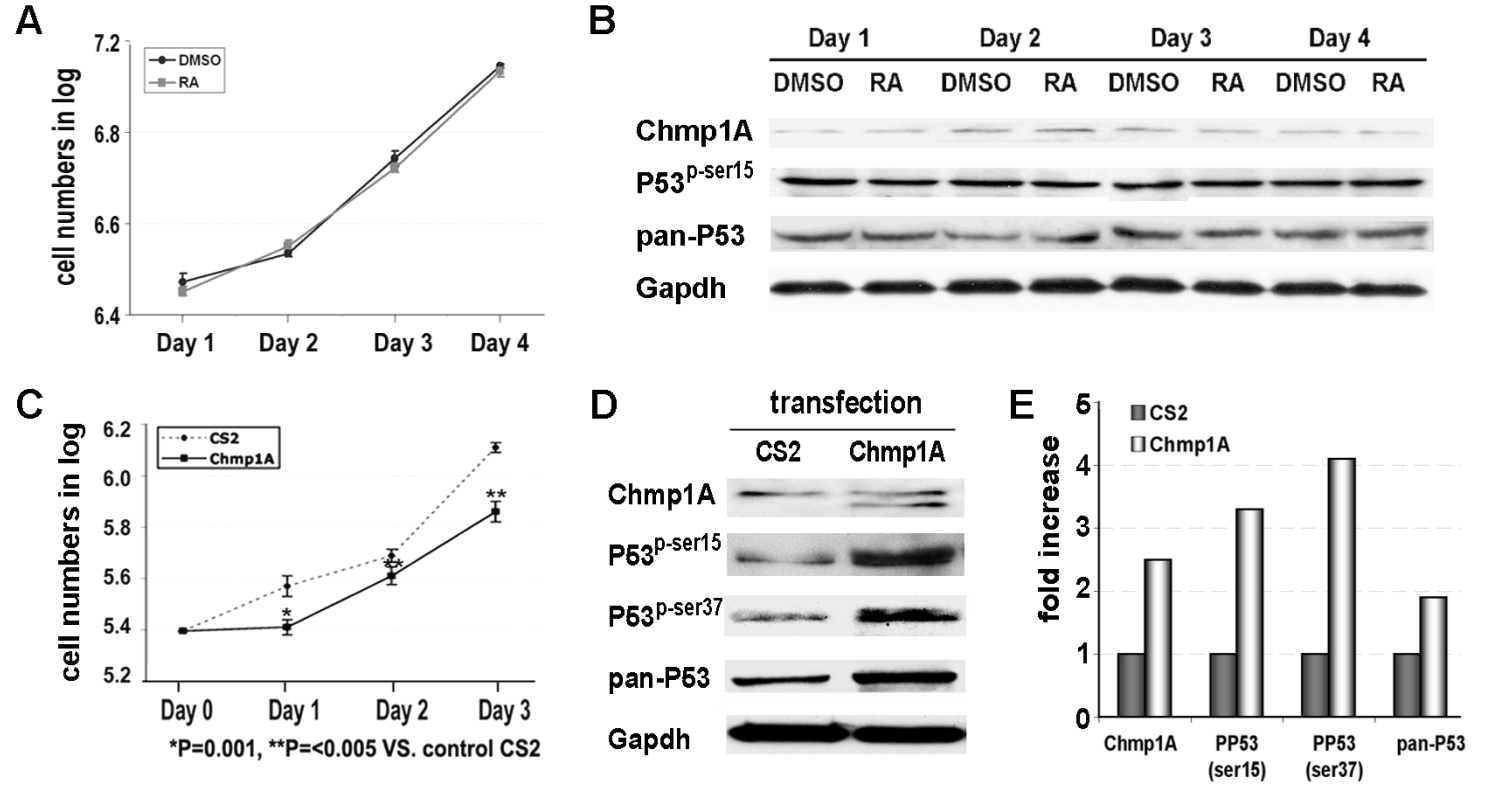
**ATRA resistant CRL-2151 cells did not show changes in Chmp1A, P53 or phospho-P53 upon ATRA treatment but showed growth inhibition and an increase in the expression of these proteins upon Chmp1A over-expression**. (A) CRL-2151 cells were counted and equal numbers of cells were seeded in 10 cm tissue culture dishes. Next day cells were treated with control DMSO or ATRA. The following 4 days, cells were counted and plotted for the graph. Black line with circle represents DMSO treated and gray line with square represents ATRA treated. Notice that there is no significant change in growth between DMSO and ATRA treated cells. (B) The cells were processed for Western blot analysis following lysis. Chmp1A, P53 and phospho-P53 protein expression showed no difference with DMSO or ATRA treated cells. Gapdh indicates the equal loading of samples. (C-E) Growth was inhibited a day after transfection with Chmp1A compared to control (C). The expression of Chmp1A, P53 and phospho-P53 (at serine 15 and 37) was increased a day after transfection with Chmp1A compare with control (D). Densitometric analysis was performed and the change of protein level was determined by setting control as "1" after corrected for Gapdh (E).

The cell lysates were prepared from cells using RIPA buffer (Boston Bioproducts Inc) plus complete mini protease inhibitor cocktail (Roche). Protein concentration of cell lysates was determined using BCA assay kit (Pierce). Equal amounts of cell lysates were subjected to 10% SDS-PAGE, and the proteins were electroblotted to nitrocellulose membranes. After blotting the membrane was incubated overnight with appropriate primary antibody (1: 400 to 1: 1000 dilution) followed by peroxidase-conjugated secondary antibody (1 to 3 hours) and was developed using an enhanced chemiluminescence kit (Amersham). To examine the effect of ATRA on protein level, we determine the density of each sample by correcting for Gapdh after densitometric analyses. On each day, the density of proteins changed by ATRA treatment was determined by setting control DMSO as "1" (Figures 1, 2, 3 and 4). Untreated cells show a similar expression level of proteins as shown in Day 1 control and data was not included in the figures.

### Confocal microscopic analysis

PanC-1 and CRL-2151 cells were seeded onto sterile glass coverslips in twelve-well plates at an approximate density of 0.5 × 10^5 ^cells/well in DMEM (Gibco) containing 10% FBS (Gibco), 100 U/ml penicillin and 100 mg/ml streptomycin (Gibco). These cells were incubated at 37°C with 5% CO_2 _(Gibco). The following day, cells were treated with vehicle DMSO or ATRA dissolved in DMSO (20 umol, final concentration). Following 24, 48, and 72 hours after ATRA treatment, cells were washed with 1× cold PBS, and fixed with 4% formaldehyde in 1 × PBS at room temperature for 30 min. Fixed cells were washed with PBS before being permeabilized in PBS/0.1% Triton X-100 (Sigma) for 5–10 min at room temperature. Following permeabilization cells were washed three times in cold PBS and incubated in blocking solution (PBS, 10% heat inactivated FBS) for 30 min. Next, the cells were incubated with Chmp1A antibody in 1 × PBS containing 5% heat inactivated serum (blocking buffer) overnight at 4°C. After being washed three times with 1 × PBS cells were incubated with anti-rabbit Alexa Fluor 488 secondary antibody (Molecular Probes). In each case secondary antisera were used at a dilution of 1:500 in blocking buffer. Cells were washed in 1 × PBS and mounted on the slide using Vectashield (Vector Laboratories, Inc. Burlingame). Confocal images were taken by using a laser-scanning microscope (Carl Zeiss LSM510) at Marshall University Imaging Core.

### Statistical Analysis

Statistical analysis was performed with Sigma Stat software using paired student *t-test *analysis. All numerical data are reported as the Mean ± SEM. P values less than 0.05 were considered statistically significant, and all P values are one tail.

## Results

### Over-expression of Chmp1A positively regulates CRBP-I expression

We performed microarray studies in HEK 293T cells to understand the functions and/or mechanisms of Chmp1A. Cellular retinol binding protein-1 (CRBP-I) was identified as one of the up-regulated genes when Chmp1A was over-expressed in HEK 293T cells. Chmp1A [GenBank: NM_002768] and CRBP-1 [GenBank: NM_002899] showed 9.44-fold and 3.46-fold increases respectively, upon Chmp1A over-expression compared with control (data not shown). Reverse transcriptase PCR (RT-PCR) was carried out to verify microarray data. As shown in Figure 1A, CRBP-1 mRNA level was increased by 3.3 fold compared to control at 31 cycles. Chmp1A mRNA expression was examined as positive control for RT-PCR which was increased 1.9 fold by Chmp1A over-expression compared to control (Figure 1A). Next, we investigated whether Chmp1A regulates CRBP-1 in PanC-1 cells. Doxycycline (dox) inducible Chmp1A stable clones were generated and tested for the induction of Chmp1A expression as described in our previous paper [22]. As previously shown Chmp1A expression was dramatically increased by dox-dependent induction of Chmp1A in two independent stable clones of PanC-1 cells. CRBP-1 protein was also increased in clone 1 and 2 by 1.4 and 1.6 folds respectively (Figure 1B). The same stable clones without dox supplement were used as controls. Since CRBP-1 is involved in retinoid metabolism and function we developed a hypothesis that Chmp1A might be involved in ATRA signaling.

### ATRA induced growth inhibition and an increase of Chmp1A, CRBP-1, P53 and phospho-P53 protein in human pancreatic ductal tumor cells

Human pancreatic ductal tumor cells, Capan-2 (well differentiated) and PanC-1 (poorly differentiated), were treated with ATRA, 20 μM final concentrations. There are two reasons we chose to use 20 μM of ATRA for the following experiments. First, we observed clear growth inhibition at 20 μM of ATRA in our experiments with various doses of ATRA (1 μM, 5 μM, 10 μM, 15 μM, and 20 μM (data not shown). Pancreatic tumor cell lines were treated with 20 μM or higher concentrations of ATRA in previously published reports [29,30]. Second, we need to use a sufficient amount of ATRA to determine the effect of Chmp1A knockdown on ATRA-mediated growth inhibition. For growth assays, cell number was counted on a daily basis for 5 days after ATRA treatment using a hemocytometer. ATRA treatment induced growth inhibition of Capan-2 and PanC-1 cells (Figures 2A and 3A, respectively) compared with vehicle DMSO treatment. Based on P-value of less than 0.05, two groups showed significant difference in growth, which is consistent with the results reported from other researchers [30-33]. To determine if Chmp1A is involved in the growth inhibition of ATRA, we performed Western blot analysis. As shown in Figures 2B and 3B, the level of Chmp1A protein was increased upon ATRA treatment in both cell lines compared to control. The increase in Chmp1A protein with ATRA treatment in Capan-2 cells was significant but appeared to be transient, observed only on day 2. However, PanC-1 cells showed an increase in Chmp1A upon ATRA treatment in all the days tested. Although it showed the highest ratio of increase in Chmp1A protein on day 1, it exhibited robust expression of Chmp1A on day 3 and 4 in both control and ATRA treated cells (Figure 3B). CRBP-1 showed similar changes in protein expression to that of Chmp1A. In Capan-2 cells, the difference in CRBP-1 expression was transient, limited to day 1 and 2 (1.36 and 1.94 fold increase each) upon ATRA treatment (Figure 2B). Capan-2 cells have a longer doubling time (about 90 hours [34]) than PanC-1 cells (about 50 hours [35]) and did not show significant growth inhibition (P > 0.05) or increase in Chmp1A expression, one day after ATRA treatment (Figure 2A). The changes in CRBP-1 in PanC-1 cells upon Chmp1A over-expression was not robust, maximum 1.3 fold increase compared to control (Figure 3B).

ATRA was shown to suppress tumor growth by regulating a known tumor suppressor P53 [36-38]. We have previously shown that Chmp1A over-expression induced an accumulation of P53 and phospho-P53 (at serine 15 and 37 position) in PanC-1 cells [22]. Thus we investigated whether ATRA controls P53 and phospho-P53 protein expression in ATRA responsive cell lines. Western blot analyses (shown in Figures 2C and 3C) demonstrated that ATRA increased the protein expression of P53 and phospho-P53 in Capan-2 and PanC-1 cells. Although Capan-2 cells did not show growth inhibition on Day 1, it showed the most significant ratio of increase in phospho-P53 (at serine 15 and 37) one day after ATRA treatment (Figure 2C). PanC-1 cells also showed an increase in phospho-P53 (at serine 15 and 37) one day after ATRA treatment (Figure 3C). The ratio of increase in phospho-P53 diminished drastically in Capan-2 cells and gradually in PanC-1 cells from Day 2 on, exhibiting robust expression of phospho-P53 (at serine 15) on Day 2 and 3 in Capan-2, and on Day 2 in PanC-1 cells, respectively. As for total P53 expression, Capan-2 cells showed a strong expression as well as an increase on Day 1. From Day 2 on, P53 expression reduced noticeably, still maintaining an increase in its expression upon ATRA treatment (Figure 2C). PanC-1 cells also showed an increase of P53 expression up to Day 3 upon ATRA treatment (Figure 3C).

### Chmp1A and P53 expression was not changed in ATRA non-responsive cells

To further determine whether the increase of Chmp1A protein is essential for the growth inhibition of ATRA we treated CRL-2151 cells with ATRA. This cell line was previously shown to be resistant to ATRA-mediated growth inhibition [39]. Consistent with previous reports ATRA did not inhibit growth of CRL-2151 cells (Figure 4A). P-values showed there was no significant differences between DMSO and ATRA treated cells (P-values; 0.18, 0.196, 0.195, and 0.23 on day 1, 2, 3, and 4, respectively in Figure 4A). In ATRA-responsive cells Chmp1A protein expression was increased upon ATRA treatment (Figures 2B and 3B). However, as shown in Figure 4B, the Chmp1A protein level was not changed in CRL-2151 cells, up to four days after ATRA treatment. In addition, as shown in Figure 4B, the expression level of total P53 and phospho-P53 (at serine 15) remained similar in ATRA treated cells compared to control cells, indicating the parallel relation between growth inhibition and accumulation of Chmp1A, P53, and phospho-P53 protein.

### Chmp1A over-expression inhibited growth of CRL-2151 cells that accompanied by an increase in P53 and phospho-P53

Above results demonstrated that CRL-2151 cells neither showed growth inhibition nor any increase in the expression of Chmp1A, P53 or phospho-P53 when they were treated with ATRA. Thus we over-expressed Chmp1A and tested whether it had any effect on the growth of CRL-2151 cells. As shown in Figure 4D, Chmp1A protein level was increased upon Chmp1A over-expression 1 and 2 days after transfection (shown here is 1 day after transfection). We did not observe an increase of Chmp1A protein 3 days after transfection (data not shown). In addition, CRL-2151 cells demonstrated growth inhibition when Chmp1A was over-expressed (Figure 2C). The growth inhibition induced by transient over-expression of Chmp1A was temporary, limited to one day but maintained the following days. Since we have shown that Chmp1A over-expression induces growth inhibition of PanC-1 cells by regulating P53 signaling [22], we investigated the expression of P53 and phospho-P53 in Chmp1A over-expressing CRL-2151 cells. As shown in Figure 4D, the protein expression of P53, and phospho-P53 (at serine 15 and 37) was increased a day after transient over-expression of Chmp1A. Densitometric analysis (Figure 4E) demonstrates significant changes in protein expression, showing 2.5, 3.3, 4.3 and 1.8 fold increase of Chmp1A, phopsho-P53 (serine 15), phopsho-P53 (serine 37), and total P53 respectively.

### Chmp1A is required for ATRA mediated growth inhibition

**Figure 5:**
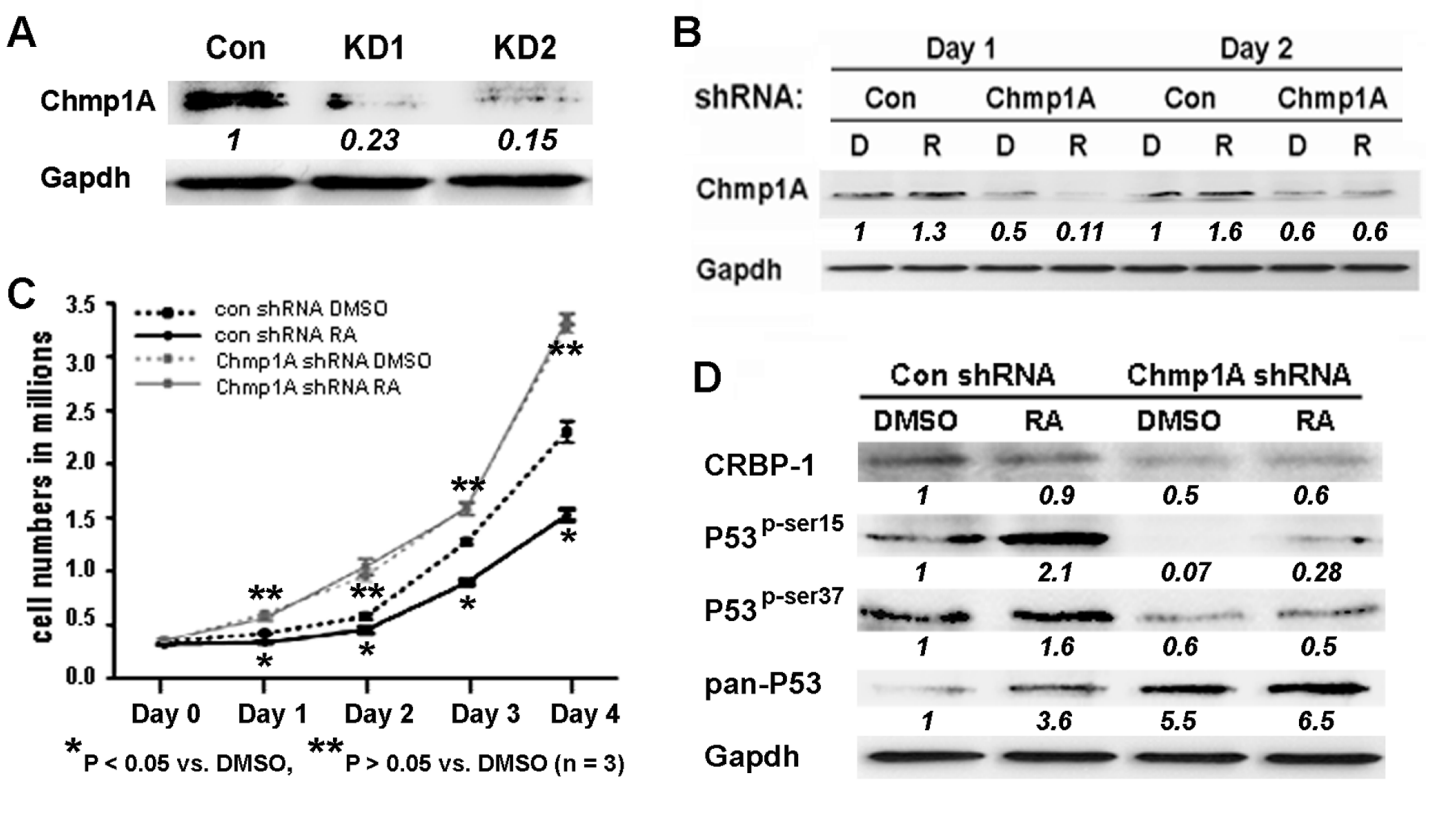
**Chmp1A knockdown abolished ATRA mediated growth inhibition and the increase of protein levels of Chmp1A, CRBP, P53 and phospho-P53**. (A) Chmp1A shRNA was cloned into shRNA RNAintro™ pSM2 retroviral vector to generate stable PanC-1 clones expressing Chmp1A shRNA. Western blot analysis demonstrated the knockdown efficiency of two colonies (KD1 and KD2). Non-silencing shRNA was used as control. (B) Chmp1A expression was reduced in Chmp1A silenced PanC-1 cells more than 50% on Day 1 and 40% on Day 2 regardless of ATRA treatment, compared with non-silencing shRNA expressing cells that were treated with vehicle DMSO. ATRA treatment increased Chmp1A expression slightly. (C) Silencing of Chmp1A induced growth promotion in the presence or absence of ATRA, compared with control cells that were treated with either DMSO or ATRA. ATRA did not have an effect on growth upon stable expression of Chmp1A shRNA compared to control shRNA. (D) Control shRNA expressing cells showed an increase in P53 and phospho-P53 at serine 15 and 37 without exhibiting an increase in CRBP-1. However, Chmp1A shRNA expressing cells exhibited a decline in CRBP-1 and phospho-P53 expression at serine 15 and 37 when compared with control shRNA expressing cells that were treated with DMSO. ATRA increased P53 expression when Chmp1A was silenced, however, Chmp1A depletion did not decrease P53 expression. D: DMSO, R: ATRA

Next we investigated whether Chmp1A is required for the growth inhibition of ATRA. We generated stable clones that express shRNA to knockdown Chmp1A in PanC-1 cells. Before we used the stable knockdown clones for growth assays, we tested the knockdown efficiency by Western blot analysis. Knockdown stable clone 1 and 2 (KD1 and KD2) showed a significant decrease of Chmp1A protein (Figure 5A), 77% and 85%, respectively, compared to a non-silencing shRNA expressing control clone. Since stable clone 2 showed greater knockdown efficiency we used these clone of cells for the following growth and Western blot analyses.

We treated cells stably expressing either control or Chmp1A shRNA with ATRA (or DMSO as control) to test whether Chmp1A was required for growth inhibition of ATRA signaling. Similar to non-treated cells, the non-silencing shRNA-expressing cells exhibited significant growth inhibition upon ATRA treatment (black solid line), compared with DMSO treatment (black dashed line, P < 0.05) (Figure 5C). On the other hand, the cells that stably express Chmp1A shRNA promoted cell growth in the presence of ATRA or DMSO. The growth promotion by Chmp1A knockdown was obvious in the DMSO treated cells (gray dashed line) as well as in the ATRA treated cells (gray solid line) compared with control. Importantly, ATRA treated PanC-1 cells that express Chmp1A shRNA revealed the same growth pattern as DMSO treated cells demonstrating that ATRA signaling is not translated to growth inhibition in the absence of Chmp1A (P > 0.05). In addition, the growth was promoted in both ATRA and vehicle treated PanC-1 cells that express Chmp1A shRNA compared to the cells that express non-silencing shRNA. Our data indicates that Chmp1A knockdown mediates growth promotion, and that Chmp1A is indispensable for the growth inhibition of ATRA signaling.

### The knockdown of Chmp1A diminished the ATRA mediated increase in protein expression level of Chmp1A, CRBP-1, and phospho-P53

We have shown that Chmp1A expression is increased upon ATRA treatment in PanC-1 cells (Figure 3B). Thus we tested whether Chmp1A expression was changed upon ATRA treatment in Chmp1A depleted PanC-1 cells. In the control shRNA-expressing cells, Chmp1A expression was increased slightly compared with control upon ATRA treatment (1.3 and 1.6 fold increase on Day 1 and 2, respectively) (Figure 5B). However, in the Chmp1A shRNA-expressing cells, Chmp1A expression was decreased on both Day 1 and 2 compared to that of control, from 1 to 0.5 (DMSO) and 0.11 (RA) and from 1 to 0.6 (DMSO) and 0.6 (RA) on Day 1 and 2 each, demonstrating the knockdown efficiency of Chmp1A. In addition, Chmp1A protein level was not increased upon ATRA treatment in Chmp1A depleted cells compared to control, confirming the requirement of Chmp1A in ATRA mediated growth inhibition.

Next we tested whether Chmp1A knockdown affected the protein expression of CRBP-1, total P53 and phospho-P53 at serine 15 and 37. The changes in CRBP-1 protein made by Chmp1A knockdown followed by ATRA treatment were somewhat complicated. CRBP-1 protein was increased upon ATRA treatment on Day 1 (data not shown) in control cells, which was decreased a little on Day 2. However, its expression went down 50% when Chmp1A was silenced, which increased slightly upon ATRA treatment on Day 2 (Figure 5D). As for P53 and phospho-P53, we have noticed significant changes 2 days after ATRA treatment only. In control shRNA-expressing cells, ATRA treatment elevated P53 and phospho-P53 expression that was similar to what we have observed from non-treated PanC-1 cells (compare Figure 5D to Figure 3C, Day 1 or Day 2). In Chmp1A depleted cells, the knockdown of Chmp1A did not abolish the ATRA mediated increase in total P53 expression. However, phospho-P53 expression was decreased upon DMSO or ATRA treatment compared with DMSO treated control shRNA expressing cells, from 1 to 0.07 (DMSO) and 0.28 (RA) at serine 15, and from 1 to 0.6 (DMSO) and 0.5 (RA) at serine 37 each.

### Nuclear expression of Chmp1A is important for ATRA mediated growth inhibition

**Figure 6:**
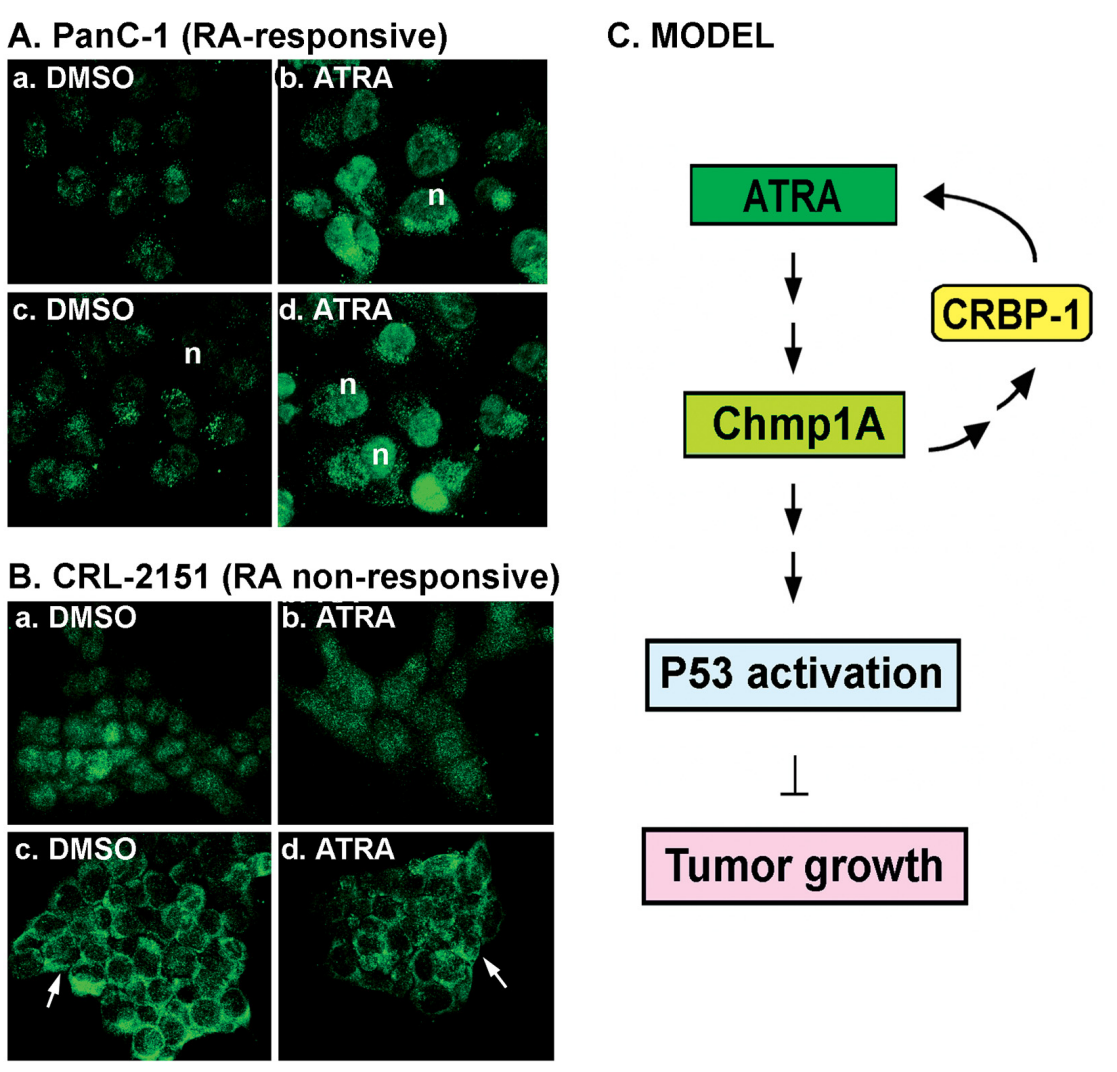
**Chmp1A was translocated to the nucleus in ATRA responsive cells but to the membrane in ATRA resistant cells upon ATRA treatment (A and B), and Model (C)**. (A) In the presence of DMSO, Chmp1A expression was modestly detected both in the nucleus and cytoplasm in PanC-1 cells (a, c). However, Chmp1A protein expression became robust in the presence of ATRA, especially in the nucleus (n in c, d). (a, b) and (c, d) is one and two days after vehicle or ATRA treatment, respectively. (B) Chmp1A was initially distributed ubiquitously in CRL-2151 cells in the presence of DMSO or ATRA (a, b). From Day 3 on, however, Chmp1A protein was mainly detected and remained at the membrane (arrows in c, d) in both DMSO and ATRA treated cells. (a, b) is for Day 2 and (c, d) is for Day 3 after DMSO and ATRA treatment, respectively. (C) Model: Chmp1A mediates growth inhibition of ATRA signaling. Chmp1A positively regulates the expression of CRBP-1. In turn, CRBP-1 controls the activity of ATRA via regulating the storage and metabolism of retinol A. ATRA treatment produces an increase in the expression level of Chmp1A in the nucleus, which leads to the accumulation of total and 'active' P53 resulting in a decrease in cell proliferation.

ATRA is known to exert its effect via interacting with its nuclear receptors [3,40]. It is possible that ATRA regulates cellular growth by enhancing the nuclear localization of Chmp1A since Chmp1A is distributed in the nucleus [19]. We tested this hypothesis by investigating the sub-cellular distribution of Chmp1A in the presence of vehicle DMSO or ATRA. Confocal microscopic analysis demonstrated that Chmp1A expression was ubiquitous with robust staining in the nucleus in ATRA responsive PanC-1 cells. When cells were treated with ATRA, Chmp1A was increased especially in the nucleus compared to control (Figures 6. Ab and d compare with 6. Aa and 6. Ac). In ATRA non-responsive cells, Chmp1A expression was also ubiquitous initially and up to day 2, with or without ATRA treatment (Figures 6. Ba and 6. Bb). However, by day three, Chmp1A was translocated into the plasma membrane and remained at the membrane in both ATRA and DMSO treated cells (Figures 6. Bc and 6. Bd).

## Discussion

In this study we provide a new insight into the function and mechanism of the Chmp1A, a member of the ESCRT-III family. As shown in our model in the conclusion, Chmp1A positively regulates CRBP-1 expression. In turn, CRBP-1 augments the availability of ATRA via controlling the storage and metabolism of retinol A [7,41,42]. ATRA then increases the protein level of Chmp1A, which subsequently causes an accumulation of P53 and phopspho-P53. This model proposes a positive amplification of ATRA signaling resulting in the inhibition of tumor cell proliferation mediated in part by Chmp1A.

CRBP-I binds retinol and is thought to carry this retinoid to various enzymes for its metabolism to retinoic acid [7]. The expression of CRBP-1 is reduced in various tumors including breast [8], prostate [42], ovarian [43], and endometrial carcinomas [44]. The decrease of cytoplasmic immunoreactivity of CRBP-1 is associated with the increase of tumor grade in endometrioid carcinomas [44]. In our study, we have shown that Chmp1A positively regulated the expression of CRBP-1 at the mRNA level in HEK 293T (Figure 1A). At the protein level, however, CRBP-1 did not show significant changes either by Chmp1A over-expression (Figure 1B) or by ATRA treatment (Figures 2B, 3B and 5D) although it showed a major decrease by Chmp1A knockdown (Figure 5D). A similar observation was made previously. Treating Sertoli cells with either stimulants or inhibitors of CRBP-1 resulted in significant changes in CRBP-1 mRNA levels. However, very little changes were observed at the protein level [45], indicating that the regulation of CRBP-1 protein is not directly correlated with that of CRBP-1 mRNA. As for a mechanism, we found SPFH domain containing proteins as Chmp1A binding partners (Belogortseva and Park, unpublished). CRBP-1 protein localizes to lipid rafts, specialized membrane domains, where retinol is stored [7]. SPFH domain-containing proteins are also found in the lipid rafts of various membranes including plasma membrane and endosomes [46]. Thus it is possible that Chmp1A, by interacting with SPFH-domain containing proteins at lipid rafts of various membranes, could have an effect on CRBP-1 and retinol storage.

We examined the involvement of Chmp1A in ATRA signaling by determining the effect of ATRA on Chmp1A expression in pancreatic tumor cell lines. We chose two ATRA responsive cell lines and one ATRA non-responsive cell line for our experiments. Capan-2 and PanC-1 cells are well- and poorly-differentiated human pancreatic ductal tumor cells, respectively, whose growth was inhibited by ATRA treatment. Capan-2 cells have longer doubling time compare to PanC-1 cells and the longer doubling time was reflected in ATRA-mediated growth inhibition (compare Figure 2A to 3A). CRL-2151 is a mouse pancreatic acinar tumor cell line that did not show growth inhibition upon ATRA treatment. Our results on ATRA-mediated growth inhibition of pancreatic tumor cells are consistent with the already published reports [30,33,39,47]. However, the increase of Chmp1A upon ATRA treatment in two ATRA responsive cell lines is a novel finding.

Our observation that ATRA increased P53 and phospho-P53 expression also agrees with several reports demonstrating that ATRA inhibits tumor growth by regulating P53 expression and activity [37,48]. This data is also consistent with our previous report [22], demonstrating that Chmp1A functions as a tumor suppressor in pancreatic tumor *in vitro *and *in vivo *in part by regulating P53. The activation and stabilization of P53 are thought to be mediated by post-translational modification of protein, with phosphorylation being the major event [49]. Although the exact function of specific phosphorylation events remains controversial, evidence indicates that they contribute to both stabilization and activation of P53. Phosphorylation of P53 at ser15 and apparently at 37 is due to activation of ATM [50]. Recent study indicates that ATRA activates ATM, but the mechanism underlying ATRA mediated activation of ATM remains unclear. It was shown previously that Chmp1A has a strong effect on chromatin structure [19]. Thus, the potential explanation in the light of our data may be that ATRA mediated Chmp1A expression can lead to changes of chromatin structure and subsequent ATM activation.

We tested whether Chmp1A is required for ATRA-induced growth inhibition by knockdown Chmp1A expression using shRNA. Non-silencing shRNA expressing PanC-1 cells exhibited a similar growth inhibition to non-treated PanC-1 cells when treated with ATRA compared to control (Figure 5C, black lines, compare with Figure 3A). However, Chmp1A shRNA expressing PanC-1 cells that greatly reduced Chmp1A protein level did not show any difference in growth with ATRA treatment as shown in Figure 5C. ATRA treated cells showed the same growth pattern as control DMSO treated cells. Moreover, Chmp1A knockdown resulted in growth promotion of pancreatic ductal tumor cells in the presence or absence of ATRA relative to cells expressing control shRNA. These results suggest that Chmp1A is essential for growth inhibition by ATRA and agree with our previous data demonstrating that Chmp1A inhibits tumor growth independent of ATRA in human pancreatic cancer cells [22].

Previously, we have shown that over-expression of Chmp1A leads to the accumulation of P53 and phospho-P53 at serine 15 and 37 [22]. The same stable clones were used to test Chmp1A and CRBP-1 expression by Chmp1A over-expression (Figure 1B). Based on these experiments, we speculated that P53 and phospho-P53 expression would be decreased when Chmp1A expression is depleted. When Chmp1A is depleted, phospho-P53 expression did go down at both serine 15 and 37 positions, compared with control (Figure 5D). As for total P53, silencing of Chmp1A did not decrease its expression on Day 2. Since it takes considerable time for shRNA to exert its effect fully, we speculate that P53 expression is still high in Chmp1A shRNA-expressing cells. However, it is possible that the level of phospho-P53, not total P53, might be important in Chmp1A-mediated growth regulation. Interestingly though, ATRA induced an increase of P53 and phospho-P53 in both control and Chmp1A shRNA expressing cells. As shown in Figure 5A, Chmp1A silenced cells still express small amounts of Chmp1A that could respond to ATRA, which relates to the increase of P53 and phospho-P53 expression in Chmp1A shRNA-expressing cells. We also used Chmp1A small interfering RNA (siRNA) to silence Chmp1A transiently, and obtained similar results in growth and protein expression in Chmp1A (data not shown).

The model (Figure 6C) is further supported by our experiments with ATRA resistant cells. Since human pancreatic acinar tumor cell lines are not available, we used mouse pancreatic acinar tumor cells for this assay. We hypothesized that ATRA non-responsive cells should not show any difference in Chmp1A protein levels. Our data indeed demonstrated that the expression of Chmp1A did not change upon ATRA treatment. In addition, we did not observe any change in the protein expression of P53 or phospho-P53. We further investigated whether transient over-expression of Chmp1A could make ATRA-resistant cells respond to signals for growth inhibition. CRL-2151 cells showed growth inhibition followed by an increase in P53 and phospho-P53 expression. These results strongly demonstrate that Chmp1A is essential for ATRA-mediated growth inhibition, and that Chmp1A also functions independently of ATRA even though it's employing the same downstream target, P53, for growth inhibitory effects. ATRA-independent functions of Chmp1A in human pancreatic ductal tumor and embryonic kidney (PanC-1 and HEK 293T respectively) were described in our previous paper in detail [22].

We also investigated whether ATRA had any effect on the nuclear localization of Chmp1A. In the absence of ATRA, Chmp1A was expressed ubiquitously in the cytoplasm and the nucleus (Figure 6) in both ATRA-responsive and non-responsive cells. However, Chmp1A protein had stronger nuclear localization upon ATRA treatment in ATRA responsive cells. In the ATRA non-responsive cells, addition of ATRA resulted in translocation of Chmp1A to the plasma membrane. These results indicate that nuclear localization of Chmp1A might be important for its ability to regulate cell proliferation through ATRA-signaling activity. Since ATRA responsive PanC-1 cells also showed significant increase in cytoplasmic expression of Chmp1A upon ATRA treatment (Figures 6Ab and 6Ad), we can't rule out the involvement of cytoplasmic Chmp1A in ATRA mediated growth inhibition.

## Conclusion

In this study, we provide novel mechanisms by which certain tumors are more sensitized to ATRA treatment than others. Our study indicates that not only the level of, but also the nuclear localization of Chmp1A is important in the mediation of growth inhibition of ATRA signaling, partly through the positive regulation of CRBP-1 (Figure 6C, Model). By developing methods to mimic Chmp1A functions, our data could lead to development of therapeutic reagents targeted to treat both ATRA sensitive and resistant tumors.

## Competing interests

The authors declare that they have no competing interests.

## Authors' contributions

JL designed, performed experiments, and participated in drafting the manuscript; BO, KW and HN participated in performing experiments and evaluating data; NB generated and purified Chmp1A antibody, participated in design experiments and discussion of the results; RN participated in the design of the study and revision of the manuscript; GB performed and analyzed microarray analyses; AD performed confocal microscopic analyses; PM conceived of the study and participated in its design and coordination, and critically revised the manuscript. The authors read and approved the final manuscript.
